# Impact of Chikungunya on Health Related Quality of Life Chennai, South India

**DOI:** 10.1371/journal.pone.0051519

**Published:** 2012-12-12

**Authors:** Vidya Ramachandran, Muniyandi Malaisamy, Manickam Ponnaiah, Kanagasabai Kaliaperuaml, Selvaraj Vadivoo, Mohan Digambar Gupte

**Affiliations:** 1 Training Division, National Institute of Epidemiology, Indian Council of Medical Research, Chennai, Tamil Nadu, India; 2 National Institute for Research in Tuberculosis, Indian Council of Medical Research, Chennai, Tamil Nadu, India; Institute of Neuroepidemiology and Tropical Neurology, France

## Abstract

**Background:**

Chikungunya Virus (CHIKV) infection affects large populations and leads to prolonged and debilitating pain affecting health related quality of life (HRQoL). We assess the impact of CHIKV on HRQoL of clinical CHIKV (C-CHIKV) patients in a suburban locality of Chennai City, South India. Further, we determined factors associated with clinical recovery among C-CHIKV patients.

**Methods:**

We followed-up 403 of 425 adult C-CHIKV cases identified during an outbreak. On the basis of a reassessment of their current clinical status through self-reporting, we categorized them as ‘clinically recovered’ (n = 308) or ‘not recovered’ (n = 95). In the absence of base-line information on HRQoL, we included a comparison group of healthy normal’s recruited by frequency matching for age and sex from the neighbourhood (n = 308). We conducted a comparative cross-sectional study of these three groups and estimated HRQoL scores using SF-36 questionnaire. We tested the differences in the median scores by Kruksall Wallistest. We identified factors associated with ‘recovery’ as compared to not-recovery by calculating Adjusted Odds Ratio (AOR) and 95% Confidence Intervals through multiple regression analysis.

**Results:**

As compared to ‘normals’, we observed a 20 and five-fold reductions in HRQoL scores for C-CHIKV patients ‘not recovered’ and ‘recovered’ respectively. Differences in HRQoL scores for all the domains were statistically significant between three groups (p<0·001). Younger age, male, absence of rashes, affliction of less than five types of joints and two weeks of joint swelling were significantly associated with recovery. HRQoL scores improved with time among those ‘clinically recovered’.

**Conclusion:**

This study provides evidence for sharp reductions in quality of life not only during active C-CHIKV associated illness but also for several months after clinical recovery compared to healthy normals. This has implications for developing intervention programmes in countries with high risk of CHIKV outbreaks.

## Introduction

Chikungunya is endemic in parts of Africa, South East Asia and the Indian sub-continent. In India, the disease was first reported and isolated in Calcutta in 1963 [1]. Thereafter, Chikungunya Virus (CHIKV) infection reappeared in late 2005 with large outbreaks being reported from the States of Andhra Pradesh, Maharashtra, Orissa, Karnataka, Tamil Nadu, Madhya Pradesh and Gujarat [2]. An estimated 1·38 million people across Southern and Central India developed symptomatic disease [3]. During 2006, Tamil Nadu reported over 100,000 cases from 31 districts, including Chennai, the capital city, being the worst affected [4]. Our group had investigated outbreaks of Chikungunya in parts of Andhra Pradesh and Tamil Nadu in South India [5].

CHIKV can damage collagen and alter connective tissue metabolism in cartilage and joints to produce severe acute arthritis. Several studies have reported persistent clinical manifestations mainly arthralgia for several months after acute infection, suggesting the possibility of chronic forms of CHIKV infection [6]. The disease is usually self-limiting and resolves with time. While the disease is generally non-fatal it often affects large populations and causes considerable pain and misery, with the possibility of affecting the quality of life. Clinical manifestation including persistent arthralgia and depression several months after CHIKV infection have been previously reported by several researchers [7]–[8].

WHO has outlined the need to measure Health Related Quality of Life (HRQoL) mainly due to the broadening concept of measuring health status, beyond traditional indicators such as mortality and morbidity. HRQoL measures the impact of a disease on daily activities, behaviour of patients, their perceived health and functional state [9]. The HRQoL scores measured by the Short Form Health Survey [SF-36] assume importance for various reasons. In the context of CHIKV, physical as well as psychological domains need to be measured. This may not be possible through some of the available instruments (such as Stanford Health Assessment Questionnaire) which concentrate mainly on medical and physical outcomes. SF-36 has the advantage of measuring eight different domains and two summary health domains. Hence, SF-36 is more suited in measuring HRQoL among clinical CHIKV (C-CHIKV) patients.

In May-June 2006, our team at the National Institute of Epidemiology (NIE), Indian Council of Medical Research (ICMR) investigated CHIKV outbreak in Gouriepet, a suburban locality near Chennai, Tamil Nadu [6]. All cases fulfilled our case definition (C-CHIKV) during the outbreak which was laboratory confirmed to be Chikungunya. All cases were diagnosed by a clinician who specialized in internal medicine. Totally 415 adults and 110 children met the case definition (for C-CHIKV) of occurrence of fever and joint pain among residents of that locality during May-June 2006. The overall attack rate was 22% [6].

In the reviewed literature, there are no studies using SF-36 questionnaire to measure the HRQoL of C-CHIKV patients in India. In view of this, the outbreak setting offered an opportunity to determine the impact of CHIKV on HRQoL of C-CHIKV patients using the SF-36 questionnaire and identify factors associated with clinical recovery among C-CHIKV patients.

## Methods

### Study Setting

We conducted this study in Gouripet (2006 population: 2,649), Avadi, a suburb of Chennai, Tamil Nadu, South India. Of these residents, 44% were males and 27% were children below 15 years. Most of the houses (90%) were semi-*pucca* (single room) structures without in-house water supply. The local administration was supplying water through water tankers sonce in 3 to10 days. This compelled the need to store water to meet daily requirements. On an average each household stored between 8–12 containers of water. A government urban health facility provides free out-patient care whereas private facilities provide both in and outpatient care for Gouripet residents.

### Study Participants

**Figure 1:**
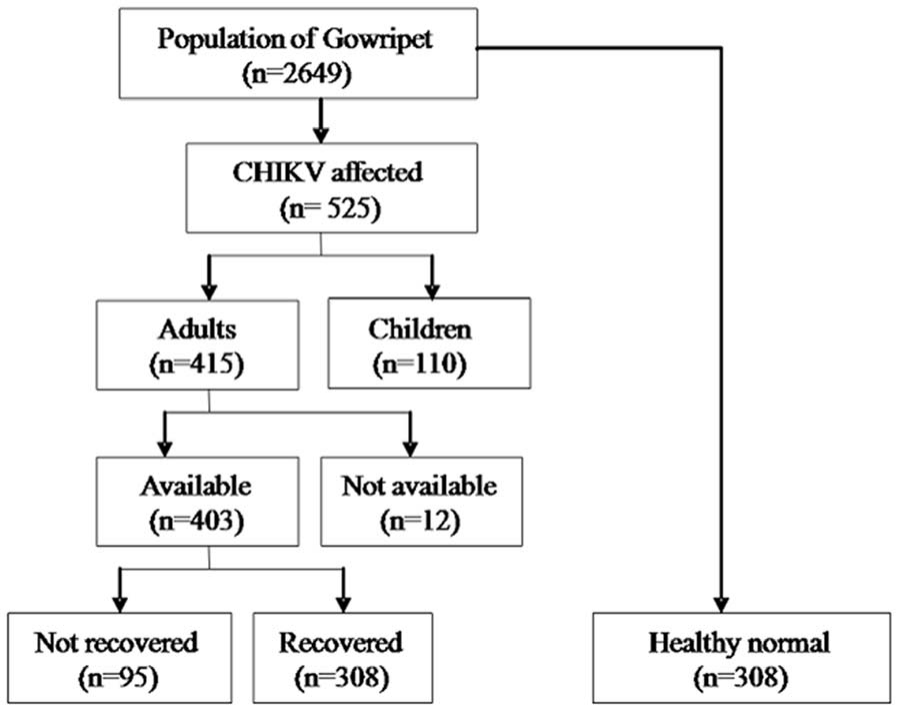
Selection of study participants.

We followed all C-CHIKV cases identified during the CHIKV outbreak who were available and willing to participate. After five months of the outbreak, we re-assessed the clinical status of all the cases based on self-reporting and classified them in to two groups. Cases without apparent signs of clinical illness were classified as ‘clinically recovered’ and cases reporting joint pain during the follow-up period were classified as ‘not recovered’. Since we did not have any base-line data for these two study groups, we recruited a third group of healthy population from the local neighbourhood for comparison (Figure-1). This group of ‘normals’ were defined as apparently healthy persons who did not meet C-CHIKV case definition during or after the outbreak, based on self-reporting and were frequency matched for age and sex. Among the eligible individuals who satisfied this definition, those available and willing to participate were included.

We excluded children, since SF-36 was meant for measuring HRQoL among adults only.

### Data Collection

We ascertained through self-reporting that all of the study participants were not treated for any illness at the time of the survey. We used SF-36, a score-based questionnaire with 36 items covering eight independent and two summary health domains [10]. These included impairment of physical and social activities, role limitations due to health problems, bodily pain, general mental health, due to emotions, vitality and general health perceptions [11]–[12]. SF-36 was translated into the local language. Using SF-36, the HRQoL was measured at five months from the date of onset of illness for all the C-CHIKV patients. For ethical reasons, all C-CHIKV patients who had not recovered were followed at six-weekly intervals until they reported that they were clinically free of all signs and symptoms. They were referred to the local health facility for treatment. However, HRQoL was not measured during six-weekly visits as this was only a single cross-sectional assessment.

To identify factors associated with ‘recovery’, we interviewed and collected data on demographic (age, sex, family size) and socio-economic characteristics (education, income). We collected reported clinical manifestations of early and late occurrence. These details included duration of joint pain, number of types of joints affected, duration of fever and joint swelling. During the interviews, we verified from the patients about past history of arthritis and arthritic pain prior to the onset of C-CHIKV illness.

All the field investigators were trained and data collection instruments were pilot-tested in the field. The interview teams were supervised by trained supervisors during data collection and every fifth interview schedule was cross-checked by them. All the data were keyed in twice by two independent data entry operators.

### Data Analysis

Based on the data collected, a score was assigned for all components on a scale of 100 using the formula recommended for SF-36. After the scores had been calculated for each domain, the scores for the two summary domains viz., physical well being component score and mental well being component score, were computed as per the SF-36 procedures [10], [13]. While ‘0’ score indicates worst quality of life, and 100 indicates best quality of life [14].

We assessed the HRQoL scores for all three groups and examined the distribution of the HRQoL scores for all the eight domains and two summary domains. To measure the impact of CHIKV on HRQoL scores of C-CHIKV patients, we compared the median differences of HRQoL scores using Kruksall Wallis Test for comparing three groups since the distribution of HRQoL scores were skewed. A p value of ≤0.05 was considered statistically significant.

To determine the factors associated with recovery, we calculated Crude and Adjusted Odds Ratios (OR) their 95% Confidence Intervals (CI) through univariate and multiple regression analysis respectively. The potential factors influencing recovery from illness identified in the study were: age (above and below mean value), gender, educational level, family size (above and below median), presence of rashes, number of types of joints affected (above and below median) and duration of joint swelling (above and below median). All the statistically significant variables in the univariate analysis were included in the multiple regression model. Backward elimination method was performed in order to identify factors independently associated with recovery of illness. We examined the relationship of duration of illness free days and HRQoL scores for all the domains for C-CHIKV patients who had clinically recovered using a radar plot. We used SPSS (18·0 version SPSS Inc, Chicago, IL) package for statistical analysis.

### Human Subject Protection

This study was approved by the technical advisory committee of NIE-ICMR. This study was an integral part of an emergency response to an outbreak and is covered by normal practice. This notwithstanding, we interviewed respondents after obtaining voluntary, written informed consent, assured them of confidentiality of the data collected and of their right to withdraw from the study at any time. To ensure a continuum of care, we further referred patients to the nearest health facility for treatment.

## Results

### Socio-demographic and Economic Characteristics

We recruited 97% (403/415) of adult cases identified during the outbreak (12 cases had left the area). Of these 76% (n = 308) reported ‘clinical recovery’ at the time of survey. Most of the socio-demographic characteristics were comparable between those ‘clinically recovered’ and healthy normals. Patients who had ‘not recovered’ included higher proportion of older persons, women, lower educational and income levels as compared to those clinically recovered and health normals (Table-1).

**Table 1:** Socio-demographic and economic characteristics of study population.

Characteristics	Clinically notrecovered (n = 95)	Clinically recovered(n = 308)	p-value	Healthy normal (n = 308)
Age ≤35 years	34 (36%)	192 (62%)		196 (64%)
Mean (SD)	41·9 (14·4)	33·8 (13·7)	0.001	33·9 (14·1)
Male gender	22 (23%)	139 (45%)	0.001	139 (45%)
≤ Middle school level of education	81 (85%)	221 (72%)	0.008	186 (60%)
Family size ≤4 members	56 (59%)	170 (55%)	0.519	208 (68%)
**Family income per month (USD)**				
Mean (SD) [Range]	89 (52) [20–280]	106 (103) [20–1000]	0.576	111 (98) [4–800]

### Clinical Manifestations

All C-CHIKV cases (n = 403) had fever and joint pain as ascertained at the time of acute phase of the illness during the outbreak. Mean duration of fever was 7 days (SD 11·9) with 79% reporting high fever. In addition, 65% experienced headache, 43% nausea, 44% vomiting, 11% rash and 54% joint swelling. Median duration of joint pain was 59 days (Range 3–872). None of them had reported a past history of any arthritic illness. Significantly (p<0·001) higher proportion of females (55%) experienced joint swelling as compared to males (34%). The duration of joint pain (>3 months) was significantly (p<0.001) higher for females (47%) as compared to males (21%). C-CHIKV cases who had ‘not recovered’ seemed to have experienced more serious illness compared to those who had ‘clinically recovered’ (Table-2).

**Table 2:** Signs and symptoms reported by C-CHIKV patients* according to clinical status.

Clinical characteristics	Clinically not recovered (n = 95)	Clinically recovered (n = 308)
**High grade fever**	89 (94%)	229 (74%)
Duration of fever (days) [Mean (SD)]	11·6 (21.3)	6 (6·3)
**Headache**	82 (86%)	178 (58%)
**Nausea**	62 (65%)	112 (36%)
**Vomiting**	56 (59%)	122 (40%)
**Joints affected**		
Ankle	82 (86%)	229 (74%)
Wrist	82 (86%)	241 (78%)
Knee	94 (99%)	295 (96%)
Phalanges	84 (88%)	226 (73%)
Tarsals	78 (82%)	213 (69%)
Metatarsals	55 (58%)	137 (44%)
Number of joints affected [Mean (SD)]	5 (1)	4 (2)
Duration of joint pain (days) [Mean (SD)]	347 (177)	42 (33)
**Swelling in joints**	68 (72%)	119 (39%)
Duration of swelling (days) [Mean & SD]	99 (168)	19 (18)
**Rash**	17 (18%)	28 (9%)
Duration of rash (days) [Mean (SD)]	17 (26)	7 (7)

* Includes early and late symptoms experienced by the patients.

### HRQoL Scores by Domains

The median scores for C-CHIKV patients ‘not recovered’ for various domains ranged between five for physical functioning to 60 for general health, scores for CHIK patients ‘clinically recovered’ ranged between 20 and 62 and for ‘healthy normals’ the scores were 100 and 85 respectively for the same domains. The differences in the median scores between all three groups for all the domains were statistically significant (p<0·001) (Table-3).

**Table 3:** Median HRQoL scores by group.

Domains	Clinically not recovered (n = 95)	Clinically recovered (n = 308)	Healthy normal (n = 308)
Physical functioning	5	20	100
Role physical	0	25	100
Body pain	0	22	100
Role emotional	17	33	100
Social functioning	0	25	100
Vitality	19	31	50
Mental health	35	40	40
General health	60	62	85
Summary			
Physical*	17	32	92
Mental**	21	37	72

Each domain’s score was significantly different at 5% level (p<0.001).

* Physical functioning; Role physical; Bodily pain & General health^10.^

** Role emotional; Social functioning; Vitality & Mental health^10.^

### Factors Associated with Non-recovery among C-CHIKV Patients

In the univariate analysis, factors significantly associated with -recovery among C-CHIKV affected patients at the time of survey were age ≤35 years, male, education above middle school, low or moderate grade fever, fever ≤4 days, non-affliction of small joints (ankle, phalanges, tarsals, metastarsals), affliction of ≤4 types of joints, joint swelling ≤14 days and absence of rash. In the multivariate analysis, age ≤35 years, male, absence of rash, affliction of ≤4 types of joints and joint swelling ≤14 days were significantly associated with recovery (Table-4).

**Table 4:** Factors associated with recovery among C-CHIKV patients after five months following outbreak investigation.

	Crude OR	95% CI	Adjusted OR*	95% CI
**Socio-demographic**				
**Age**				
>35 years			1·0	
≤35 years	3·0	1.8–4.9	2·6	1·6–4·4
**Gender**				
Female			1·0	
Male	2·7	1.6–4.8	2·7	1·5–4·6
**Clinical presentation**				
**Rashes**				
Present			1·0	
Absent	2·2	1.1–4.4	2·2	1·1–4·4
**Number of joints** **affected**				
>4 joints			1·0	
≤4 joints	2·2	1.3–3.9	2.0	1·1–3·4
**Duration of joints** **swelling**				
>14 days			1·0	
≤14 days	2·9	1.7–5.0	1·9	1·1–3·3

* In the multiple regression analysis.

### Factors Influencing HRQoL

We examined the influence of several factors on HRQoL scores for eight independent domains. Age, duration of fever, multiple joint affliction, duration of joint pain and duration of joint swelling were significantly and negatively associated with HRQoL scores for various domains. Age and affliction of multiple joints were associated with all domains except mental health. Similarly duration of joint pain was associated with all domains except general health. While physical functioning and role emotional were associated with duration of fever, mental health and general health were associated duration of joint swelling.

### Relationship of Length of Illness Free Days and HRQoL

**Figure 2:**
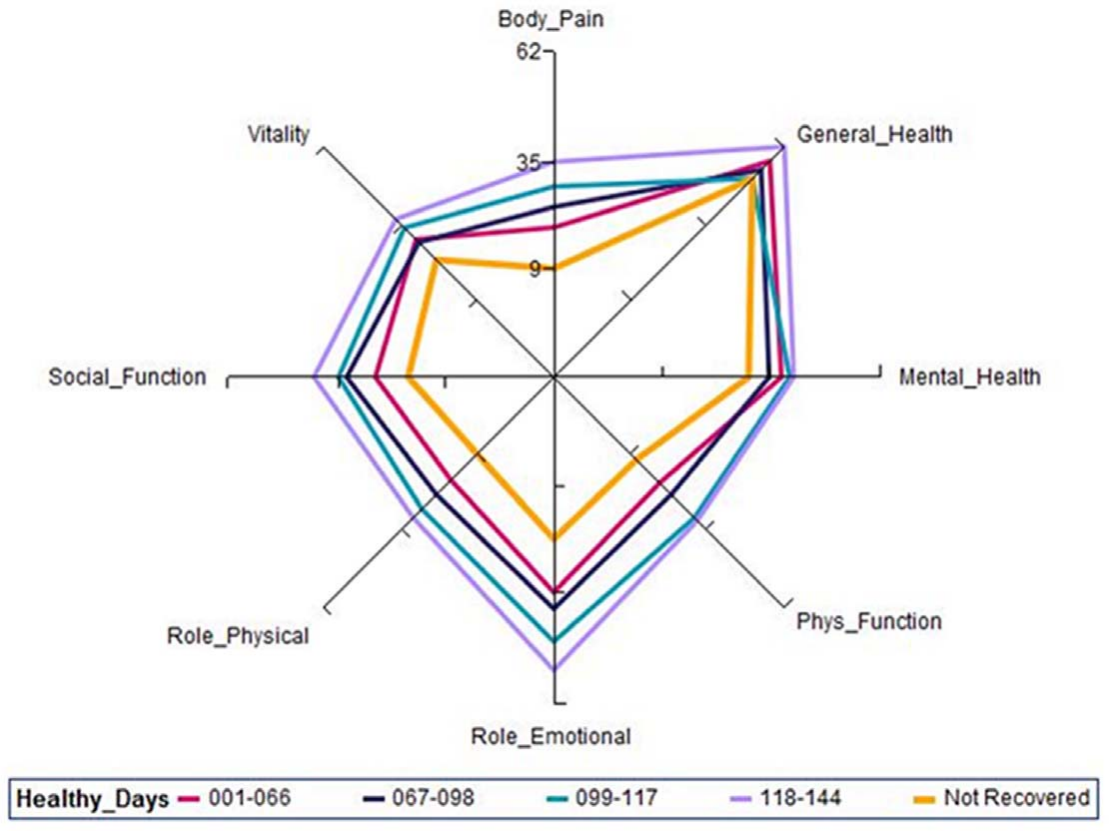
HRQoL scores of clinically recovered C-CHIKV patients according to duration of disease free days (healthy days) at five months of follow-up.

For patients who had clinically recovered at five months, we observed that within this five-month period, the recovery took place at different time points from the date of onset of illness. A radar plot depicts the relationship between HRQoL scores for all eight domains and illness free days (health days) between onset of illness and fifth month of follow-up for C-CHIKV patients ‘clinically recovered’ (Figure-2). The HRQoL scores for those C-CHIKV patients who had recovered at five months or recovered at different time points within the five months period were different. For C-CHIKV patients who recovered early, the HRQoL scores were higher than patients who recovered later. In other words, as length of disease free days increases, the HRQoL scores for many domains also increases.

## Discussion

For the first time, we report the impact of CHIKV on HRQoL measured by SF -36 among three comparison groups viz., C-CHIKV cases ‘not recovered’, C-CHIKV cases ‘clinically recovered’ and ‘healthy normals’. The key finding of our study is a greater than 20 fold reduction in HRQoL scores for domains assessing physical quality of life and a 3–5 fold reduction for other domains for C-CHIKV cases who have ‘not recovered’ compared to ‘healthy normals’. A 2–5 fold reduction is observed for most domains for C-CHIKV cases who have ‘clinically recovered’ compared to ‘healthy normals’. The significant reductions in HRQoL scores in the domains of role physical, body pain and physical functioning may be because in CHIKV infections, as in other acute illnesses such as dengue, physical debilitation may be intense for the first few days when fever persists and joint pain is typically severe necessitating hospitalization in several cases. However, unlike dengue, CHIKV infection is also characterised by a chronic phase of illness when arthralgia persists for several months to even years [15], thus explaining the sharp reductions in scores for the physical domains among C-CHIKV cases who have “not recovered”. This is inspite of ruling out any past history of arthritic illness in the C-CHIKV cases.

A study by Man-Koumba Soumahoro *et al* [8] reported only moderate reduction in the HRQoL among cases after one year following infection. This difference in the observed reductions could be due to the shorter duration of (5-months) follow-up in our study compared to the one year follow-up in the above study, suggesting that HRQoL scores for C-CHIKV patients improve with time. Significant reductions in the HRQoL scores of case patients after five months of infection observed in our study and moderate reduction in the score after one year also provides evidence for the prolonged and adverse impact of CHIKV on HRQoL of affected individuals. However, the generic instrument used by Man-Koumba Soumahoro *et al* [8] i.e., SF-12 is different from the instrument used in the present study (SF-36). This could explain the reason for observed differences between the two studies.

Our study confirms the well known inverse relationship between age and HRQoL [16]. We further documented that the duration of joint pain and number of types of joints affected are important and acted as negative correlates for HRQoL. In fact, in the Reunion Island, Sissoko and co-workers identified that older age and severe initial joint pains were important risk factors for non-recovery [17].

Both sexes were affected, with females being at significantly higher risk compared to males. This may be because in our study most of the females were home makers spending more time indoors and thus becoming more susceptible to bites from infected Aedes mosquitoes within the home. However, when the median HRQoL scores for all the domains were compared for males and females no significant differences were observed. This is inspite of the fact that the mean duration of joint pain was significantly higher for females compared to males, implying that though the severity of illness/infection may be similar in both sexes, females take longer time for recovery. This may be because most women tend go back to the household work even while they are still suffering, thus increasing the time for recovery.

The HRQoL scores for patients who had not recovered and those who had clinically recovered suggested that during active infection/disease, the severity of the illness reduces the HRQoL of the patients to the lowest level. The nearly four- fold reductions in HRQoL scores among those “clinically recovered” compared to “healthy normals” implies that ‘clinical’ recovery is not synonymous with attaining of normal HRQoL scores and also testifies to the prolonged, residual adverse impacts of the disease on the patients. This finding reinforces the need to assess HRQoL of C-CHIKV patients during active illness and after clinical recovery at periodic intervals till they attain HRQoL scores that are comparable with HRQoL scores of healthy individuals. Such an assessment will provide insights about the length of time taken to attain normalcy after clinical recovery and consequently enable health programme managers plan and implement a continuum of appropriate interventions that ultimately safeguard health and assure an optimal quality of life for affected individuals.

### Conclusions

Our study provides evidence for heavy reduction in the HRQoL scores associated with CHIKV not only during acute illness but also for several months after clinical recovery from illness. This has serious implications since the disease affects large populations with ensuing cumulative debilitating effect on HRQoL. Issues related to HRQoL of patients need further research using clinical reviews to better understand the natural history, pathogenesis and long-term impact of persisting manifestation following CHIKV. This has important implications for developing intervention programmes in countries with high risk of CHIKV outbreaks.
